# How gender theories are used in contemporary public health research

**DOI:** 10.1186/s12939-017-0712-x

**Published:** 2018-03-20

**Authors:** Anne Hammarström, Gunnel Hensing

**Affiliations:** 1 Department of Public Health and Caring Sciences, Uppsala University, Uppsala, Sweden; 2 Department of Public Health and Community Medicine, Section of Social Medicine, Sahlgrenska Academy, University of Gothenburg, Gothenburg, Sweden

**Keywords:** Gender, Theories, Public health, Categorical thinking, Methods

## Abstract

**Background:**

Public health research often focuses on gender differences within certain diagnoses, but so far research has failed to explain these differences in a satisfactory way. Theoretical development could be one prerequisite for moving beyond categorical thinking. The aim of this paper was to analyse how gender theories have been used in public health research in relation to various methodological approaches.

**Method:**

Six special issues of gender research with public health relevance (comprising 33 papers in total) were identified from a search of PubMed and Web of Science, spanning a 10-year period. The papers were analysed inductively through posing questions to the text.

**Results:**

Gender theories were used in eight different ways: 1. to test hypotheses, 2. integrate theories, 3. develop gender concepts and models, 4. interpret findings, 5. understand health problems, 6. illustrate the validity of other theories, 7. integrated into a gender blind theory, as well as to 8. critique of other gender theories. The strategies applied seemed independent of the health aspects of the papers. However, the methodologies were of importance, indicating that both theoretical papers and papers using qualitative methodologies used almost all available strategies, while papers using quantitative empirical research used a limited number of strategies.

**Conclusions:**

This study contributes to identifying how gender theories are used in contemporary public health research, which can help researchers move beyond a categorical understanding of gender in health research.

**Electronic supplementary material:**

The online version of this article (10.1186/s12939-017-0712-x) contains supplementary material, which is available to authorized users.

## Background

International research shows that substantial gender differences exist within certain major diagnoses, such as hypertension [1] and depression [2]. However, researchers have so far failed to explain these differences in a satisfactory way. [1–4] This might in part be related to the fact that public health studies often favour empirical research while the researchers lack strong theoretical frameworks [5]. Thus, much research is devoted to replicating previously known gender differences in health status rather than to explain their origin, which also might contribute to the preservation of gender stereotypes.In order to find better explanations for gender differences in health status we need to use gender-related concepts, models or theories, yet there is still a lack of development of gender theories in public health research [6].

The lack of gender theoretical analyses in the final report of the influential Commission of Social Determinants in Health [7] (CSDH) has been highlighted as a major problem. The commission was set up by the WHO in 2005 in order to marshal evidence on what could be done to reach equity in health from a global perspective. The commission organised itself in knowledge networks in various areas, among them one called ‘Women and Gender Equity’ network. The chapter from this network in the final report from the CSDH [7] has been heavily criticised by feminist researchers. While acknowledging its focus on gender on a structural level, embedded in social relations of power, Bates et al. [8] criticised the chapter for not adequately using contemporary gender theories. Their main critique is directed towards equating gender with women, which leads to inadequate attention on how gender interacts with other dimensions of social stratification and neglect of gender in relation to men’s health. In addition, Raewyn Connell [9] has criticised the report for its lack of gender theoretical approach and for its categorical thinking about gender. Categorical thinking implies that men and women are viewed as fixed, unproblematic categories and that gender becomes reduced to a statistical difference between men and women [9].

As gender researchers in public health, we recognise the difficulties of moving beyond the statistical quantification of differences between men and women, which is common in epidemiological research. Gender theoretical development is central in the process of moving beyond categorical thinking. In previous research, we developed a tool (i.e., a set of questions) to help researchers move from research that focuses on gender differences to more complex gender theoretical research [10, 11]. In gender research, there is a need for questioning the dominating epistemology in medicine as well as for gender theoretical development including power analyses as well as analyses of differences within the group of men/women [10].

In previous research, we have also clarified our use of gender related concepts in order to advance the development of gender theories [6, 12]. Since we are often asked how gender theories can be used in public health research, we believe an important next step towards a comprehensive theory of gender in public health would be to study ***how*** gender theories are actually used in contemporary public health research. To the best of our knowledge, this is the first study about how theories can be used in public health research.

## Methods

The aim of the paper was to analyse how gender theories have been used in public health in relation to various methodological approaches.

### Material

A purposeful sampling of public health papers expected to have a strong gender theoretical base was chosen to provide relevant and rich information. As gender theoretical development is difficult to publish in traditional medical or public health journals [12], we decided to select special issues in which the included papers have greater opportunities to develop gender theories.

A literature search was performed in May 2016 in PubMed and Web of Science with the search terms: ((gender[Title]) OR (femini*[Title]) OR (sex*[Title]) OR (masculin*[Title])). The search was limited to English language papers published since 2005, and in order to find special issues we first searched for editorials.

The inclusion criteria required editorials to be for a special issue with public health relevance, defined in a broad way as dealing with population health issues (e.g., health status, health behaviour and health care experiences). As such, editorials introducing special issues about clinical treatment of patients were excluded.

In total, we found 282 editorials in PubMed and (partly overlapping) 339 editorials in Web of Science (*n* = 621). The sex* search term yielded many excluded publications dealing with sexuality without any public health focus. In addition, most of the editorials were not related to any special issue and were therefore excluded. When the editorials did introduce a special issue, many of these issues had a narrow clinical focus that was out of scope for our analyses of public health research. We identified 13 special issues with public health relevance. All papers in these special issues were read and analysed in relation to two inclusion criteria:

1. Public health relevance, defined in a broad way as dealing with population health while excluding research about clinical treatment of patients
2. Use of gender theories (as described under the Analyses heading).

No papers in the following seven special issues fulfilled these two criteria and were therefore excluded: Issues Ment Health Nurs. 2012 Dec;33(12) Maturitas 2011 Mar;68(3), Fam Med 2011 Mar;43(3), Violence Against Women. 2010 Feb;16(2): Int J Tuberc Lung Dis. 2008 Jul;12(7), Women Health 2007;46(1), Soc Sci Med. 2007 Mar;64(5).

The following six special issues contained at least one paper and therefore fulfilled our inclusion criteria (33 papers in total):

I. Current Sociology 2009–7 papers
II. Social Science and Medicine (intersectionality) 2012–16 papers
III. Scandinavian Journal of Work Environment and Health 2005 – 1 paper
IV. International Review of Psychiatry 2011–1 paper
V. Ergonomics 2012-– 1 paper
VI. Social Science and Medicine (masculinities and suicidal behaviour) 2012–7 papers

These Roman numerals will be used below for referring to the various special issues. A reference list of all included papers is available in Additional file 1.

#### Analyses

We read all papers (*n* = 33) featured in the selected special issues and used the following two ways for identifying the gender theories utilised therein:

1. Identification of text about gender theories (such as gender constructions, masculinities, femininities, gender relations, gendered power structures, intersectionality). In addition, theories often used in gender research (e.g., Foucault’s theories of bio power and power and knowledge [13], as well as embodiment theories [14, 15]) were included when the focus of the paper was on gender.
2. Identification of text with references to known gender theoreticians (such as Judith Butler, Raewyn Connell, Olena Hankivsky, Nancy Krieger, Beverly Skeggs, Sylvia Walby etc.). Such text was scrutinised for gender theories as in the first point.

The analysis of how gender theories were used was developed inductively in two steps. First, we read each paper with a focus on how gender theories were used and identified two overarching ways: a., in order to explain a health phenomenon or, b., health phenomena were used to illustrate that the gender theory was valid (see Table 1). The first author reread all papers again with a focus on finding other ways of using gender theories and discussed the findings with the second author. Each paper was coded in relation to the four questions in Table 1. During this procedure we identified several other ways of using gender theories.

**Table 1:** Step 1 Analyses of gender theories

1. Which gender concepts and theories are used in relation to health issues? Which are the main theories and which are the alternatives?
2. What aspect(s) of health does the main theory address and seek to explain?
3. How are the theories used?
a. Is the theory used to explain health problems? If yes, make a deeper analysis about HOW. How is the theory applied to/developed in relation to health/illness?
b. Is the health problem used in order to show that the theory is valid?
4. Which methodological approach was used in the paper?

Second, these various ways of using gender theories were translated into specific questions (Table 2a and b). All papers were coded once again in order to answer ‘yes’ or ‘no’ to the questions in Table 2a and b (see Additional file 2). Initially, each paper was also coded in relation to the following two questions: ‘Other ways of using gender theories: How?’ and ‘Other reflections of the use of gender theories in the paper?’ The answers to these questions were translated into existing or new questions in Table 2a and b.

**Table 2:** Step 2 of the analysis

a. The questions about how gender theories were used in scientific papers		
	Yes	No
1. Does the paper introduce gender theories in order to test hypotheses?		
2. Does the paper integrate gender theories in various parts?		
3. Does the paper develop gender concept and models?		
4 Does the paper interpret empirical findings from gender theories?		
5 Does the paper use gender theories in order to explain health problems		
6 Does the paper use the health problem in order to illustrate that the theory is valid/ the implications of the theory		
7 Do the authors use/ integrate gender theories in traditional gender blind theories?		
8 Do they use gender theory to criticise other feminist theories?		
Other ways of using the gender theories: HOW?		
Other reflections of the use of gender theories in the paper?		
b. Strategies reformulated from the questions above		
Gender theories were used to: 1. to test hypotheses 2. integrated in various parts of the paper 3. to develop gender concept and models 4. to interpret empirical findings 5. to understand health problems 6. to illustrate the validity of theories with health status as example 7. integrated in traditional gender blind theories 8. to criticise other feminist theories		

Three of the 33 papers were editorials and mainly contained a description of which gender theories were used in the papers of the special issue. As such, these editorials were not very interesting to code in relation to the questions in Table 2a and b and so they were not included in that analysis.

In Table 3 we summarised our main findings from Additional file 2. Here we have translated the questions about how gender theories were used into strategies. Information is also provided about which gender theories, health aspects, and methodological approach (i.e., distinguishing between editorials, theoretical papers, empirical papers using qualitative methods, and empirical papers using quantitative methods) were used in each paper. Finally, in the Table 4 we give a short background description of the gender theories that were most often used in our analysed papers.

**Table 3:** Overview over the gender concepts and theories used in the papers

Ref^a^	Which gender concepts and theories are used in relation to health issues	What aspect(s) of health does the main theory address	How are the theories used? (Strategy 1 to 8 in Table 2b)	Methods
1	CURRENT SOCIOLOGY Intersectionality Constructions of masculinities/ femininities	“Health” unspecified	Not relevant to code - editorial	Editorial
2	Post-modern theories Materiality of bodies, social construction of differences	Health care unspecified	2, 8	Theoretical
3	Relational theory of gender Gender constructions	Coronary heart disease	2, 4	Review of qualitative methods
4	Intersectionality	General practitioner visits	2, 4	Empirical qualitative
5	Intersectionality Hegemonic masculinity Deconstruction of binary categories	HIV test	1, 2	Mixed method empirical
6	Gender constructions, gender relations Gender identities	Sexual reproductive health issues	2, 4, 5	Empirical qualitative
7	Foucault – biopower, power and knowledge	Male menopause	4, 5	Empirical qualitative
	SOCIAL SCIENCE MEDICINE			
8	Relational, intersectional, and biosocial approaches	Health unspecified, autism	Not relevant to code - editorial	Editorial
9	Gender bias Social constructivism	Autism	1, 2, 6	Theoretical
10	Relational theory of gender	Anorexia	2, 6, 8	Theoretical
11	Sex and gender in interaction Embodiment	Behavioral and biological differences in early ages.	2, 3	Theoretical
12	Intersectionality	HIV, mental illness	2, 6	Theoretical
13	Masculinity Intersectionality	Drug abuse	2, 6	Empirical qualitative
14	Gender constructions and agency	Sexuality	2, 3, 4, 5	Empirical qualitative
15	Gendered embodiment Sexualised medical surveillance Diagnose as a frame of gendered interpretations/expectations	CAH congenital adrenal hyperplasia	2,7	Review
16	Early feminist critic of surrogacy Liberal feminism defended surrogacy Intersectionality	assisted reproductive technologies	2, 3	Empirical qualitative
17	Gender system Hegemonic masculinity	Public health messages	2, 4, 5	Empirical qualitative
18	Gender socialization of role theories Gender relational approach Doing gender	Health behaviour	2, 5, 8	Empirical qualitative
19	Intersectionality Double and Triple jeopardy hypothesis Masculinities, Femininities Relational selves	Mental health	1, 2, 5	Empirical quantitative
20	Intersectionality	Long-term illness	1, 2	Empirical quantitative
21	Criticism against dichotomies and differences Sex and gender entanglement (sex/gender) Intersectional	Human health unspecified	2, 5, 7, 8	Theoretical
22	Post-modern and post-colonial feminism Hegemonic masculinities Gender mainstream	International health unspecified	2, 8	Empirical qualitative
23	Feminist intersectional framework	Health and well-being unspecified	2, 4, 5, 6	Empirical qualitative
24	Gender order	“Health” unspecified	2	Quantitative methodological
25	Social constructivism	Mental health	2, 5	Review
26	Multiple role theory, Role stress theory	Musculoskeletal disorders and emotional exhaustion	2, 4, 5	Quantitative empirical
27	Masculinities, gender as performative, critics a sex-difference framework and essentialism	Suicide	Not relevant to code - editorial	editorial
28	Gendered identities and practices, masculinity crisis, objectivist rather than constructivist understanding, gendered scripts of suicide, (does not mention power, more focus on sociology)	Suicide	2	Qualitative, social autopsy, empirical
29	Masculinity, hegemonic masc., powerful males, biological distinction between male and female human beings, male power, patriarchal, gendered life circumstances (violence, sexuality, supply family)	Suicide	2, 4, 5	Quantitative empirical
30	Differences within the group of men (age) Criticizes that masculinities are used for explaining all male behaviour (cause and effect model), models of masculinity are not applicable on boys, backlash against feminism	Suicide	2	Qualitative empirical
31	Constructions of masculinities, Criticism of the construction of men as one single group and of Western dualism of body and mind.	Suicide	2, 5, 8	Qualitative empirical
32	Masculinities (identities, roles, norms, hegemonic), (intersecting with class etc.), agency within structure, gendered power relations, less socially connected,	Suicide	2, 4, 5	Qualitative empirical
33	Gender relations, construction of masculinities, gendered life circumstances, gender roles	Suicide	2, 4, 5	Review of qualitative papers

^a^References refer to Additional file 1

**Table 4:** Background descriptions of the most often used gender theories

A crucial concept in the development of gender research has been ‘doing gender’ or ‘constructions of gender’, which in a basic sense means creating social and behavioral differences (that do not exist) between men and women [24]. Poststructuralist theory [25] has developed as a critique of such an essentialist approach, that is, the tendency to regard differences between men and women as constant and unchangeable. Foucault [13] was an important inspiration for this development.
As an influential critic against both categorical thinking and the lack of materialism of post structuralism, Raewyn Connell has developed the relational theory of gender [9]. According to her, the “relational theory usually understands gender as multidimensional: embracing at the same time economic relations, power relations, affective relations and symbolic relations; and operating simultaneously at intrapersonal, interpersonal, institutional and society-wide levels” [26]. Connell defines gender order as the structure of gender relations in a given society at a given time [26].
Theories about gender constructions were developed by Raewyn Connell [27, 28] and others from feminist theories of patriarchy and debates over the role of men in transforming patriarchy. Connell defines hegemonic masculinity as the “pattern of practice (i.e., things done, not just a set of role expectations or an identity) that allowed men’s dominance over women to continue” [27] (page 832).
Intersectionality is based on the underlying assumption of heterogeneity within the groups of ‘men’ and ‘women’ and recognises that individuals are defined by multiple, intersecting dimensions, such as gender, class, ethnicity, (dis)ability, sexuality, age, etc. [6]. This approach was first developed by Crenshaw [29] and later by Olena Hankivsky [30, 31]and others as a critique against the dichotomous way of dividing gender into ‘men’ and ‘women’, without analysing differences within the group of men and within the group of women.

## Results

The six special issues differed in relation to both their focus and theoretical approach. The health outcomes were specific in three of them; mental health (IV), ergonomics (V) and suicide (VI), while unspecific in the others. The gender theoretical focus was specified in some of the special issues: as relational, intersectional and biosocial (II), to focusing on masculinities (VI). In Current Sociology (I), the theoretical point of departures was specified as the sociological understanding of gender and health (health as gendered, unpacking gender as social category, the health needs of various gender identities, medicalization and the sociology of the body). Gender differences (without references to gender theories) in occupational health as well as in ergonomics were in focus in two special issues (III, V), while special issue IV dealt with more general biological and epidemiological differences in mental health.

### How were the theories used?

As illustrated by the eight questions in Table 2b, we inductively identified the following eight ways of using gender theories in the reviewed material (the number of times each strategy was used is denoted in parentheses):

Gender theories were used to:

1. test hypotheses (4)
2. be integrated in various parts of the paper (29)
3. develop gender concept and models (3)
4. interpret empirical findings (11)
5. understand health problems (14)
6. illustrate the validity of theories with health status as an example (5)
7. be integrated in traditional gender blind theories (2)
8. critique other gender theories (7).

Jewkes & Morell [16] could be used as a good example in relation to several of the above identified strategies. Their Introduction was permeated by gender theories (strategy 2) such as the framework of gender and power, hegemonic masculinity, and social constructions of gender within power hierarchies. With qualitative methods, the paper inductively developed gendered concepts (strategy 3) in terms of various constructions of femininities in relation to HIV risk practices grounded in a framework of agency within structure. The results were interpreted (strategy 4) and discussed in relation to gender theories (patriarchy, male power, contextualised femininities etc.). Thus, strategies 2, 3 and 4 were identified. In addition, strategy 5 was also evident since gender theories were used to understand HIV risk practices.

The paper by Jordan-Young [17] is notable since it was the only one using strategy 7, i.e., how researchers integrate gendered aspects/theories into a gender blind medical theory. Jordan-Young made a critical feminist analysis of the congenital adrenalin hyperplasia (CAH) hypothesis (that steroids in utero shape “brain gender” and gender ‘atypical’ behaviour in humans) and expanded the ‘tunnel vision’ of the hypothesis to include gendered aspects of living with CAH (such as medical interventions, living with atypical genitals, how physical manifestation of CAH becomes entangled with lived gendered experiences).

Strategy 4 (to use theories in order to interpret findings) is related to (but not the same as) strategy 2 (integrating theories). Strategy 2 includes papers in which the gender theories were integrated in the whole paper or only in some part of the paper, often in the Introduction section. Even though it was not the aim of this paper to analyse how strongly the identified gender theoretical approaches contributed to developing gender theories, these eight ways do say something about how pronounced the gender theoretical approach was. The focus on gender theories is probably strongest when the aim of a paper is involved, such as in Muñoz-Laboy et al. [18], which includes gender theories about gender systems and constructions of masculinities in relation to health needs and risk behaviour. Furthermore, papers that develop gender concepts/ theories are often theoretically advanced.

From a public health perspective, strategy 5 (to use gender theories in order to understand health aspects) is more interesting than strategy 6 in which health issues are used to validate a gender theories, while the authors seem less interested in developing the health focus. To use gender theories in order to critique other gender theories (strategy 8) is part of the theoretical development within the field.

Some of the strategies were dependent on the methodology; to test a hypothesis (strategy 1) is primarily performed in quantitative research while concepts are mainly developed with qualitative methods.

Table 3 summarises which gender theories, health aspects and methodologies that were used in the analysed papers.

The table shows that many of the papers had an ambitious use of various gender theories as well as of a wide range of strategies. The strategies were used, seemingly independent of the health aspects. However, they differed in relation to the various methodological approaches. Almost all possible strategies were used in theoretical papers (strategies 1, 2, 3, 5, 6, 7 and 8) and in papers using qualitative methods (strategies 2, 3, 4, 5, 6 and 8). Theoretical papers do not have empirical results and therefore cannot use strategy number 4. Empirical qualitative papers seldom test hypotheses, and strategy number 1 was not used in these papers. Quantitative papers had the most limited use of strategies – they only used four of the eight strategies (number 1, 2, 4 and 5). Even though it is important to do studies that are stratified by women and men, there is a risk of the simplified use of categorical and biological explanations. Thus, the methodology might lead to a reductionist approach unable to move beyond stereotypical and dualistic understandings of women, men and health.

In relation to health aspects, Table 3 shows that seven of the included papers had a vague focus on ‘health’ (e.g., writing about health as an unspecified topic), while eight papers focused on reproductive health issues, and seven were about suicide. Surprisingly, the strong theoretical focus on gender in several of the papers was accompanied by an almost complete lack of theoretical reflections about the outcome (i.e., the health aspects) of the papers. In spite of the fact that the editorial of Current Sociology 2009 underlined the importance of ‘looking at health as a gendered issue in sociological research’ [19], there was a lack of awareness about gender theories in relation to the health outcome in most of the papers therein. A more distinct focus on the outcome as gendered was found in some of the papers of the special issues on suicide. For example, Oliffe et al. [20] stated that depression was a “decidedly unmasculine ailment”, seen as a woman’s disease and certain masculinities were constructed as risk behaviour including suicidal behaviour. In addition, another of the papers [21] used theories in writing about the mental health outcome, in this case the gendered construction of symptoms of mental health. Jordan-Young’s paper also included gendered analysis of the outcome in relation to living with congenital adrenal hyperplasia [17].

There was a notable lack of focus (apart from one paper about coronary heart disease) on major public health problems like cancer, diabetes, cardiovascular diseases and health behaviours. Furthermore, none of the studies were devoted to better understanding prevention and health promotion from a gender perspective.

Table 4 provides a brief background to the most commonly used gender theories in the analysed papers.

Interestingly, all of the gender theories featured in the reviewed papers were developed outside the field of public health/medicine, mainly in social science. In addition, the theories mirror the wide range of perspectives that exist in gender research.

#### Implications of our findings

The categorical thinking in public health research about gender-related topics should be met with gender theoretical development. With this paper we hope to both inspire and demonstrate to researchers how to develop gender theories in public health research. We do this by providing a set of strategies for using gender theories in relation to various methodological approaches (both qualitative and quantitative, as well as mixed methods approaches) within the broad field of public health.

As a study initiating this approach, we consider our findings to also be useful in education. Public health practitioners have diverse backgrounds. In medical and health science professions, a binary understanding of women and men from a mainly biological point of view is usually predominant in education. However, most public health problems need to be addressed combining bio-psycho-social perspectives. Gender theories open up new perspectives for how to understand women and men in their various contexts, including gendered structures and norms. An important aspect in developing teaching material on gender and health is the inclusion of various gender theories. Our paper has the potential to be an important aid in such training, addressing both the approaches that are currently most used, as well as highlighting how new theoretical perspectives can be applied in research.

A problem within the field of public health research, is that theories are generally underdeveloped. However, there is an increasing theoretical interest in health promotion. This interest has resulted in evidence that public health interventions developed within an explicit theoretical framework are more effective than those performed without a theoretical base [22, 23], Thus, theories are useful in explaining why certain interventions are successful while others are not. Our study identified no gender theoretical paper within the field of health promotion. Through gender theoretical development, the effects of public health interventions can be improved for both men and women with various backgrounds.

All of the gender theories identified in this review were developed outside the field of public health. In addition, the surprisingly weak theoretical interest in the health outcomes, in combination with the lack of focus on the most common public health diseases, may reflect the lack of public health researchers within our analyses. Among the 33 papers analysed, only four had authors from the field of public health. Consequently, there is an urgent need for public health researchers to engage in gender theoretical research. Our findings provide a stepping stone to further studies within the field.

In this paper we have addressed gender theories, but our approach regarding the interpretation of science, and the development of theories in science, may well be used in analyses of intersectional theories that focus on power dimensions other than gender (e.g., race, sexuality, socioeconomic status) as well as in the few available models/ frameworks in public health, such as social causation and health selection.

## Conclusions

This is the first study to analyse how gender theories have been used in public health research. We identified eight ways of using gender theories within the field of gender and health. The methodologies were of importance for the strategies; both theoretical papers and papers using qualitative methodologies used almost all available strategies while papers using quantitative empirical research used a more limited range of strategies. Thus, there is a potential for quantitative studies to improve their use of gender theories.

Nancy Krieger [5] argues for the need of theories as a way of improving public health research, to avoid errors, spark new ideas, and enable us to be critical of studies. She argues “…by making conscious use of epidemiological theory and having informed debates over the different theoretical perspectives in play we stand a better chance of producing epidemiological knowledge truly useful for preventing disease, promoting health equity and improving people’s health.” And to that we would add: …to move beyond categorical thinking in relation to research on gender and health.

## Additional files

Additional file 1: List of references. (DOCX 38 kb)

Additional file 2: Empirical results of analyses of step 1 and step 2 (see Tables 1 and 2a, b). Additional file 1 shows the list of references. (DOCX 76 kb)
