# Supplementary material for: How gender theories are used in contemporary public health research
Source: Int J Equity Health. 2018 Mar 20;17:34. doi: 10.1186/s12939-017-0712-x (PMC5859645; doi:10.1186/s12939-017-0712-x)
Supplement: Supplementary file 1 — List of references. (DOCX 38 kb) [file 12939_2017_712_MOESM1_ESM.docx]

Additional file 1 LIST OF REFERENCES

**2009 Current Sociology**

1. Annandale, E., Riska, E., 2009. New Connections: Towards a Gender-Inclusive Approach to Women’s and Men’s Health. Current Sociology 57, 123–133.
2. Kuhlmann, E., 2009. From Women’s Health to Gender Mainstreaming and Back Again Linking Feminist Agendas and New Governance in Healthcare. Current Sociology 57, 135–154.
3. Emslie, C., Hunt, K., 2009. Men, Masculinities and Heart Disease A Systematic Review of the Qualitative Literature. Current Sociology 57, 155–191.
4. Edwards, J., Roekel, H. van, 2009. Gender, Sexuality and Embodiment Access to and Experience of Healthcare by Same-Sex Attracted Women in Australia. Current Sociology 57, 193–210.
5. Coleman, C., Lohan, M., 2009. Men Who Have Sex with Men and Partner Notification in Ireland Beyond Binary Dualisms of Gender and Healthcare. Current Sociology 57, 211–230.
6. Mufune, P., 2009. The Male Involvement Programme and Men’s Sexual and Reproductive Health in Northern Namibia. Current Sociology 57, 231–248.
7. Vainionpää, K.J., 2009. Finnish Commercial Web-Based Information on Male Menopause and Male Hormone Therapy. Current Sociology 57, 291–307.

**2012 Social Science and Medicine**

1. Springer, K.W., Hankivsky, O., Bates, L.M., 2012a. Gender and health: Relational, intersectional, and biosocial approaches. Social Science & Medicine 74, 1661–1666.
2. Cheslack-Postava, K., Jordan-Young, R.M., 2012. Autism spectrum disorders: Toward a gendered embodiment model. Social Science & Medicine 74, 1667–1674.
3. Connell, R., 2012. Gender, health and theory: Conceptualizing the issue, in local and world perspective. Social Science & Medicine 74, 1675–1683.
4. Fausto-Sterling, A., Coll, C.G., Lamarre, M., 2012a. Sexing the baby: Part 1 – What do we really know about sex differentiation in the first three years of life? Social Science & Medicine 74, 1684–1692.
5. Hankivsky, O., 2012. Women’s health, men’s health, and gender and health: Implications of intersectionality. Social Science & Medicine 74, 1712–1720.
6. Hansen, H., 2012. The “new masculinity”: Addiction treatment as a reconstruction of gender in Puerto Rican evangelist street ministries. Social Science & Medicine 74, 1721–1728.
7. Jewkes, R., Morrell, R., 2012. Sexuality and the limits of agency among South African teenage women: Theorising femininities and their connections to HIV risk practises. Social Science & Medicine 74, 1729–1737.
8. Jordan-Young, R.M., 2012. Hormones, context, and “Brain Gender”: A review of evidence from congenital adrenal hyperplasia. Social Science & Medicine 74, 1738–1744.
9. Markens, S., 2012. The global reproductive health market: U.S. media framings and public discourses about transnational surrogacy. Social Science & Medicine 74, 1745–1753.
10. Muñoz-Laboy, M., Perry, A., Bobet, I., Bobet, S., Ramos, H., Quiñones, F., Lloyd, K., 2012. The “knucklehead” approach and what matters in terms of health for formerly incarcerated Latino men. Social Science & Medicine 74, 1765–1773.
11. Reczek, C., Umberson, D., 2012. Gender, health behavior, and intimate relationships: Lesbian, gay, and straight contexts. Social Science & Medicine 74, 1783–1790.
12. Rosenfield, S., 2012. Triple jeopardy? Mental health at the intersection of gender, race, and class. Social Science & Medicine 74, 1791–1801.
13. Sen, G., Iyer, A., 2012. Who gains, who loses and how: Leveraging gender and class intersections to secure health entitlements. Social Science & Medicine 74, 1802–1811.
14. Springer, K.W., Mager Stellman, J., Jordan-Young, R.M., 2012b. Beyond a catalogue of differences: A theoretical frame and good practice guidelines for researching sex/gender in human health. Social Science & Medicine 74, 1817–1824.
15. Tolhurst, R., Leach, B., Price, J., Robinson, J., Ettore, E., Scott-Samuel, A., Kilonzo, N., Sabuni, L.P., Robertson, S., Kapilashrami, A., Bristow, K., Lang, R., Romao, F., Theobald, S., 2012. Intersectionality and gender mainstreaming in international health: Using a feminist participatory action research process to analyse voices and debates from the global south and north. Social Science & Medicine 74, 1825–1832.
16. Weber, L., Hilfinger Messias, D.K., 2012. Mississippi front-line recovery work after Hurricane Katrina: An analysis of the intersections of gender, race, and class in advocacy, power relations, and health. Social Science & Medicine 74, 1833–1841.

**2009 Scandinavian journal of work and environmental health**

1. Härenstam, A., 2009. Exploring gender, work and living conditions and health - suggestions for contextual and comprehensive approaches. Scandinavian Journal of Work, Environment & Health 35, 127–133.

**2010 Int Rev Psychiatry**

1. Andermann L. Culture and the social construction of gender: mapping the intersection with mental health. Int Rev Psychiatry. 2010;22(5):501-12. doi: 10.3109/09540261.2010.506184.

**2012 Ergonomics**

1. Ahlgren C, Malmgren Olsson EB, Brulin C. Gender analysis of musculoskeletal disorders and emotional exhaustion: interactive effects from physical and psychosocial work exposures and engagement in domestic work. Ergonomics. 2012;55(2):212-28.

**2012 SOCIAL SCIENCE & MEDICINE 2012  Volume: 74   Issue: 4**

1. : Canetto SS, Cleary A. Men, masculinities and suicidal behaviour. Soc Sci Med. 2012 Feb;74(4):461-5. doi: 10.1016/j.socscimed.2011.11.001. Epub 2011 Dec 1. PubMed PMID: 22189083. Editorial
2. Scourfield J, Fincham B, Langer S, Shiner M. Sociological autopsy: an integrated approach to the study of suicide in men. Soc Sci Med. 2012Feb;74(4):466-73. doi: 10.1016/j.socscimed.2010.01.054. Epub 2010 May 24. PubMed PMID: 20646811.
3. Adinkrah M. Better dead than dishonored: masculinity and male suicidalbehavior in contemporary Ghana. Soc Sci Med. 2012 Feb;74(4):474-81. doi:10.1016/j.socscimed.2010.10.011. Epub 2010 Oct 29. PubMed PMID: 21075496.
4. Mac An Ghaill M, Haywood C. Understanding boys': thinking through boys, masculinity and suicide. Soc Sci Med. 2012 Feb;74(4):482-9. doi:10.1016/j.socscimed.2010.07.036. Epub 2010 Aug 26. PubMed PMID: 20833461.(teori I sista stycket före conclusion)
5. Cleary A. Suicidal action, emotional expression, and the performance of masculinities. Soc Sci Med. 2012 Feb;74(4):498-505. doi:10.1016/j.socscimed.2011.08.002. Epub 2011 Aug 23. PubMed PMID: 21930333.
6. Oliffe JL, Ogrodniczuk JS, Bottorff JL, Johnson JL, Hoyak K. "You feel like you can't live anymore": suicide from the perspectives of Canadian men who experience depression. Soc Sci Med. 2012 Feb;74(4):506-14. doi: 10.1016/j.socscimed.2010.03.057. Epub 2010 May 24. PubMed PMID: 20541308.
7. Alston M. Rural male suicide in Australia. Soc Sci Med. 2012 Feb;74(4):515-22. doi: 10.1016/j.socscimed.2010.04.036. Epub 2010 May 25. PubMed PMID: 20541304.
