# Supplementary material for: How gender theories are used in contemporary public health research
Source: Int J Equity Health. 2018 Mar 20;17:34. doi: 10.1186/s12939-017-0712-x (PMC5859645; doi:10.1186/s12939-017-0712-x)
Supplement: Supplementary file 2 — Empirical results of analyses of step 1 and step 2 (see Tables 1 and 2a, b). Additional file 1 shows the list of references. (DOCX 76 kb) [file 12939_2017_712_MOESM2_ESM.docx]

**Additional file 2. Empirical results of analyses of step 1 and step 2 (see Table 1 and 2). Appendix 1 shows the list of references.**

**CURRENT SOCIOLOGY 2009**

| **1.** | **Annandale E Riska E 2009** |
| --- | --- |
| 1 | Multiple femininities and masculinities and Intersectionality  Social constructivism , Social causation, Agency |
| 2 | Refers to the included papers such as men’s health, medicalization |
| 3 | Editorial - Step 2 not analysed |

| **2** | **Kuhlmann 2009** |
| --- | --- |
| 1 | Post-modern theories, materiality of bodies, social construction of differences |
| 2 | Women’s health movement, gender mainstreaming of health care |
| 3 | Aim is to link experiences from women’s health movement to gender mainstreaming and feministic approaches. Neither a nor b. |

| Step 2 | Yes | No |
| --- | --- | --- |
| 1. Does the paper use gender theories in order to test hypothesis? |  | X |
| 2. Does the paper integrate gender theories in various parts? | X |  |
| 3. Does the paper develop gender concept and models? |  | X |
| 4. Does the paper interpret empirical findings from gender theories? |  | X |
| 5. Does the paper use gender theories in order to understand health problems? |  | X |
| 6. Does the paper use the health problem in order to illustrate that the theory is valid/ the implications of the theory? |  | X |
| 7. Do the authors use/ integrate gender theories in traditional gender blind theories? |  | X |
| 8. Do they use gender theory to criticise other feminist theories? | X |  |

| **3** | **Emslie Hunt 2009** |
| --- | --- |
| 1 | Gender constructions  Relational theory of gender  Male norm, universal man |
| 2 | Coronary heart disease |
| 3 | Neither a nor b. |

| Step 2 | | Yes | No |
| --- | --- | --- | --- |
| 1. Does the paper use gender theories in order to test hypothesis? | |  | X |
| 2. Does the paper integrate gender theories in various parts? | | X |  |
| 3. Does the paper develop gender concept and models? | |  | X |
| 4. Does the paper interpret empirical findings from gender theories? | | X |  |
| 5. Does the paper use gender theories in order to understand health problems? | |  | X |
| 6. Does the paper use the health problem in order to illustrate that the theory is valid/ the implications of the theory? | |  | X |
| 7. Do the authors use/ integrate gender theories in traditional gender blind theories? | |  | X |
| 8. Do they use gender theory to criticise other feminist theories? | |  | X |
|  | | | |
| **4** | **Edwards, J., Roekel, H. van, 2009.** | | |
| 1 | Intersectionality  Gender bias, anti-essentialism, deconstructionism  Identity theory ..fixed versus flexible | | |
| 2 | Health care research (visit to general practitioner) | | |
| 3 | Neither a nor b. | | |

| Step 2 | Yes | No |
| --- | --- | --- |
| 1. Does the paper use gender theories in order to test hypothesis? |  | X |
| 2. Does the paper integrate gender theories in various parts? | X |  |
| 3. Does the paper develop gender concept and models? |  | X |
| 4. Does the paper interpret empirical findings from gender theories? | X |  |
| 5. Does the paper use gender theories in order to understand health problems? |  | X |
| 6. Does the paper use the health problem in order to illustrate that the theory is valid/ the implications of the theory? |  | X |
| 7. Do the authors use/ integrate gender theories in traditional gender blind theories? |  | X |
| 8. Do they use gender theory to criticise other feminist theories? |  | X |

| **5** | **Coleman Lohan 2009** |
| --- | --- |
| 1 | Intersectionality, diversities of gender and sexualities  Hegemonic masculinity  Deconstruction of binary categories |
| 2 | HIV test of partners |
| 3 | Neither a nor b. |

| Step 2 | Yes | No |
| --- | --- | --- |
| 1. Does the paper use gender theories in order to test hypothesis? | X |  |
| 2. Does the paper integrate gender theories in various parts? | X |  |
| 3. Does the paper develop gender concept and models? |  | X |
| 4. Does the paper interpret empirical findings from gender theories? |  | X |
| 5. Does the paper use gender theories in order to understand health problems? |  | X |
| 6. Does the paper use the health problem in order to illustrate that the theory is valid/ the implications of the theory? |  | X |
| 7. Do the authors use/ integrate gender theories in traditional gender blind theories? |  | X |
| 8. Do they use gender theory to criticise other feminist theories? |  | X |

| **6** | **Mufune P 2009.** |
| --- | --- |
| 1 | Gender constructions, (role theory) gender relations  Gender identities |
| 2 | Sexual reproductive health issues among men |
| 3 | The theory is used to explain health problems |

| Step 2 | Yes | No |
| --- | --- | --- |
| 1. Does the paper use gender theories in order to test hypothesis? |  | X |
| 2. Does the paper integrate gender theories in various parts? | X |  |
| 3. Does the paper develop gender concept and models? |  | X |
| 4. Does the paper interpret empirical findings from gender theories? | X |  |
| 5. Does the paper use gender theories in order to understand health problems? | X |  |
| 6. Does the paper use the health problem in order to illustrate that the theory is valid/ the implications of the theory? |  | X |
| 7. Do the authors use/ integrate gender theories in traditional gender blind theories? |  | X |
| 8. Do they use gender theory to criticise other feminist theories? |  | X |

| **7** | **Vainionpää KJ 2009** |
| --- | --- |
| 1 | Foucault – bio-power, power and knowledge. Construction of male menopause. |
| 2 | Male menopause |
| 3 | Use gender theories (Foucault) in order to explain health problems |

| Step 2 | Yes | No |
| --- | --- | --- |
| 1. Does the paper use gender theories in order to test hypothesis? |  | X |
| 2. Does the paper integrate gender theories in various parts? |  | X |
| 3. Does the paper develop gender concept and models? |  | X |
| 4. Does the paper interpret empirical findings from gender theories? | X |  |
| 5. Does the paper use gender theories in order to understand health problems? | X |  |
| 6. Does the paper use the health problem in order to illustrate that the theory is valid/ the implications of the theory? |  | X |
| 7. Do the authors use/ integrate gender theories in traditional gender blind theories? |  | X |
| 8. Do they use gender theory to criticise other feminist theories? |  | X |

**SOCIAL SCIENCE & MEDICINE 2012**

| **8** | **Editorial Springer KW, Hankivsky O, Bates LM 2012.** |
| --- | --- |
| 1 | Gender relational, intersectional approaches, gender as agential process, social construction of sex and gender, Biosocial approach |
| 2 | Refers to health-related topics in the special issue |
| 3 | Editorial. Step 2 not relevant to code. |

| **9** | **Cheslack-Postava K, Jordan-Young RM 2012.** |
| --- | --- |
| 1 | Gender bias, Social constructivism , embodiment, sex and gender in interaction (biosocial interaction), gender relations, Gender socialization, a model about gender as pervasive developmental environment |
| 2 | Autism |
| 3 | They use gender theories to discuss(explain) autism |

| Step 2 | Yes | No |
| --- | --- | --- |
| 1. Does the paper use gender theories in order to test hypothesis? | X |  |
| 2. Does the paper integrate gender theories in various parts? | X |  |
| 3. Does the paper develop gender concept and models? |  | X |
| 4. Does the paper interpret empirical findings from gender theories? |  | X |
| 5. Does the paper use gender theories in order to understand health problems? |  | X |
| 6. Does the paper use the health problem in order to illustrate that the theory is valid/ the implications of the theory? | X |  |
| 7. Do the authors use/ integrate gender theories in traditional gender blind theories? |  | X |
| 8. Do they use gender theory to criticise other feminist theories? |  | X |

| **10** | **Connell RW 2012** |
| --- | --- |
| 1 | Gender relational approaches, categorical thinking post-structuralist |
| 2 | Anorexia HIV |
| 3 | 3b Anorexia is used to show that the theory is valid |

| Step 2 | Yes | | No |
| --- | --- | --- | --- |
| 1. Does the paper use gender theories in order to test hypothesis? |  | | X |
| 2. Does the paper integrate gender theories in various parts? | X | |  |
| 3. Does the paper develop gender concept and models? |  | | X |
| 4. Does the paper interpret empirical findings from gender theories? |  | | X |
| 5. Does the paper use gender theories in order to understand health problems? |  | | X |
| 6. Does the paper use the health problem in order to illustrate that the theory is valid/ the implications of the theory? | X | |  |
| 7. Do the authors use/ integrate gender theories in traditional gender blind theories? |  | | X |
| 8. Do they use gender theory to criticise other feminist theories? | | X |  |

| **11** | **Fausto-Sterling A, Coll CG, Lamarre M 2012** |
| --- | --- |
| 1 | Sex and gender in interaction, embodiment |
| 2 | Behavioural and biological differences in early ages.. |
| 3 | Not a nor b. |

| Step 2 | Yes | | No |
| --- | --- | --- | --- |
| 1. Does the paper use gender theories in order to test hypothesis? |  | | X |
| 2. Does the paper integrate gender theories in various parts? | X | |  |
| 3. Does the paper develop gender concept and models? | X | |  |
| 4. Does the paper interpret empirical findings from gender theories? |  | | X |
| 5. Does the paper use gender theories in order to understand health problems? |  | | X |
| 6. Does the paper use the health problem in order to illustrate that the theory is valid/ the implications of the theory? |  | | X |
| 7. Do the authors use/ integrate gender theories in traditional gender blind theories? |  | | X |
| 8. Do they use gender theory to criticise other feminist theories? | |  | X |

| **12** | **Hankivsky 2012** |
| --- | --- |
| 1 | Intersectionality |
| 2 | HIV, mental illness |
| 3 | 3b uses health to illustrate that the theory is valid |

| Step 2 | Yes | No |
| --- | --- | --- |
| 1. Does the paper use gender theories in order to test hypothesis? |  | X |
| 2. Does the paper integrate gender theories in various parts? | X |  |
| 3. Does the paper develop gender concept and models? |  | X |
| 4. Does the paper interpret empirical findings from gender theories? |  | X |
| 5. Does the paper use gender theories in order to understand health problems? |  | X |
| 6. Does the paper use the health problem in order to illustrate that the theory is valid/ the implications of the theory? | X |  |
| 7. Do the authors use/ integrate gender theories in traditional gender blind theories? |  | X |
| 8. Do they use gender theory to criticise other feminist theories? |  | X |

| **13** | **Hansen 2012** |
| --- | --- |
| 1 | Masculinity  Intersectionality  Gendered addiction  Post-industrialism |
| 2 | Drug abuse |
| 3 | 3b Masculinity theories in focus – redefined masculinities as treatment for drub abuse. |

| Step 2 | Yes | No |
| --- | --- | --- |
| 1. Does the paper use gender theories in order to test hypothesis? |  | X |
| 2. Does the paper integrate gender theories in various parts? | X |  |
| 3. Does the paper develop gender concept and models? |  | X |
| 4. Does the paper interpret empirical findings from gender theories? |  | X |
| 5. Does the paper use gender theories in order to understand health problems? |  | X |
| 6. Does the paper use the health problem in order to illustrate that the theory is valid/ the implications of the theory? | X |  |
| 7. Do the authors use/ integrate gender theories in traditional gender blind theories? |  | X |
| 8. Do they use gender theory to criticise other feminist theories? |  | X |

| **14** | **Jewkes Morrell 2012** |
| --- | --- |
| 1 | Gender constructions, femininities, agency, patriarchy |
| 3 | Sexuality |
| 3 | 3a: inductive analyses of data. Uses gender concepts/theories in order to understand their findings. |

| Step 2 | Yes | No |
| --- | --- | --- |
| 1. Does the paper use gender theories in order to test hypothesis? |  | X |
| 2. Does the paper integrate gender theories in various parts? | X |  |
| 3. Does the paper develop gender concept and models? | X |  |
| 4. Does the paper interpret empirical findings from gender theories? | X |  |
| 5. Does the paper use gender theories in order to explain health problems | X |  |
| 6. Does the paper use the health problem in order to illustrate that the theory is valid/ the implications of the theory? |  | X |
| 7. Do the authors use/ integrate gender theories in traditional gender blind theories? |  | X |
| 8. Do they use gender theory to criticise other feminist theories? |  | X |

| **15** | **Jordan Young 2012** |
| --- | --- |
| 1 | A critical feminist analyses of the CAH hypothesis (as part of the brain organization theory) (for gender ‘atypical’ behavior). How physical manifestation of CAH becomes entangled with lived gendered experiences  Gendered embodiment  Sexualised medical surveillance  Negative body image  Diagnose as a frame of gendered interpretations/expectations |
| 2 | CAH congenital adrenal hyperplasia |
| 3 | 3a Uses gender theories in order to understand the findings of the review |

| Step 2 | Yes | No |
| --- | --- | --- |
| 1. Does the paper use gender theories in order to test hypothesis? |  | X |
| 2. Does the paper integrate gender theories in various parts? | X |  |
| 3. Does the paper develop gender concept and models? |  | X |
| 4. Does the paper interpret empirical findings from gender theories? |  | X |
| 5. Does the paper use gender theories in order to understand health problems? |  | X |
| 6. Does the paper use the health problem in order to illustrate that the theory is valid/ the implications of the theory? |  | X |
| 7. Do the authors use/ integrate gender theories in traditional gender blind theories? | X |  |
| 8. Do they use gender theory to criticise other feminist theories? |  |  |

| **16** | **Markens 2012** |
| --- | --- |
| 1 | Early feminist critic of surrogacy and male control over women’s bodies, exploitation of poor women  Liberal feminism defended surrogacy  Challenge gendered ideas of maternity, reinforce genetic influence  Intersectionality  Social constructivism  Framing analyses |
| 2 | ART assisted reproductive technologies. |
| 3 | Neither a nor b. |

| Step 2 | Yes | No |
| --- | --- | --- |
| 1. Does the paper use gender theories in order to test hypothesis? |  | X |
| 2. Does the paper integrate gender theories in various parts? | X |  |
| 3. Does the paper develop gender concept and models? | X |  |
| 4. Does the paper interpret empirical findings from gender theories? |  | X |
| 5. Does the paper use gender theories in order to understand health problems? |  | X |
| 6. Does the paper use the health problem in order to illustrate that the theory is valid/ the implications of the theory? |  | X |
| 7. Do the authors use/ integrate gender theories in traditional gender blind theories? |  | X |
| 8. Do they use gender theory to criticise other feminist theories? |  | X |

| **17** | **Muñoz-Laboy et al 2012** | | |
| --- | --- | --- | --- |
| 1 | Gender system  Hegemonic masculinity | | |
| 2 | Public health messages | | |
| 3 | 3a Construction of masculinities are used in order to understand if they deny their health needs. | | |
|  | |  |  |
| Step 2 | | Yes | No |
| 1. Does the paper use gender theories in order to test hypothesis? | |  | X |
| 2. Does the paper integrate gender theories in various parts? | | X |  |
| 3. Does the paper develop gender concept and models? | |  | X |
| 4. Does the paper interpret empirical findings from gender theories? | | X |  |
| 5. Does the paper use gender theories in order to understand health problems? | | X |  |
| 6. Does the paper use the health problem in order to illustrate that the theory is valid/ the implications of the theory? | |  | X |
| 7. Do the authors use/ integrate gender theories in traditional gender blind theories? | |  | X |
| 8. Do they use gender theory to criticise other feminist theories? | |  | X |

| **18** | **Reczek Umberson 2012** |
| --- | --- |
| 1 | Gender socialization (role theories)  Gender relational approach  Doing gender |
| 2 | Health behavior work |
| 3 | 3a Use gender theories in order to understand health behaviour work. |

| Step 2 | Yes | No |
| --- | --- | --- |
| 1. Does the paper use gender theories in order to test hypothesis? |  | X |
| 2. Does the paper integrate gender theories in various parts? | X |  |
| 3. Does the paper develop gender concept and models? |  | X |
| 4. Does the paper interpret empirical findings from gender theories? |  | X |
| 5. Does the paper use gender theories in order to understand health problems? | X |  |
| 6. Does the paper use the health problem in order to illustrate that the theory is valid/ the implications of the theory? |  | X |
| 7. Do the authors use/ integrate gender theories in traditional gender blind theories? |  | X |
| 8. Do they use gender theory to criticise other feminist theories? | X |  |

| **19** | **Rosenfield 2012** |
| --- | --- |
| 1a | Intersectionality  Double and Triple jeopardy hypothesis  Hegemonic, protest, black and white masculinities  Femininities  Relational selves |
| 2 | Mental health |
| 3 | 3a Uses gender theories in order to analyse and explain the distribution of mental health |

| Step 2 | Yes | No |
| --- | --- | --- |
| 1. Does the paper use gender theories in order to test hypothesis? | X |  |
| 2. Does the paper integrate gender theories in various parts? | X |  |
| 3. Does the paper develop gender concept and models? |  | X |
| 4. Does the paper interpret empirical findings from gender theories? |  | X |
| 5. Does the paper use gender theories in order to understand health problems? | X |  |
| 6. Does the paper use the health problem in order to illustrate that the theory is valid/ the implications of the theory? |  | X |
| 7. Do the authors use/ integrate gender theories in traditional gender blind theories? |  | X |
| 8. Do they use gender theory to criticise other feminist theories? |  | X |

| **20** | **Sen Iyer 2012** |
| --- | --- |
| 1 | Intersectionality |
| 2 | Long-term illness |
| 3 | Neither a nor b |

| Step 2 | Yes | No |
| --- | --- | --- |
| 1. Does the paper use gender theories in order to test hypothesis? | X |  |
| 2. Does the paper integrate gender theories in various parts? | X |  |
| 3. Does the paper develop gender concept and models? |  | X |
| 4. Does the paper interpret empirical findings from gender theories? |  | X |
| 5. Does the paper use gender theories in order to understand health problems? |  | X |
| 6. Does the paper use the health problem in order to illustrate that the theory is valid/ the implications of the theory? |  | X |
| 7. Do the authors use/ integrate gender theories in traditional gender blind theories? |  | X |
| 8. Do they use gender theory to criticise other feminist theories? |  | X |

| **21** | **Springer et al 2012** |
| --- | --- |
| 1 | Criticism against dichotomies and differences  Sex and gender entanglement (sex/gender)  Intersectional |
| 2 | Cardiovascular disease |
| 3 | 3a Use gender theories in order to understand health |

| Step 2 | Yes | No |
| --- | --- | --- |
| 1. Does the paper use gender theories in order to test hypothesis? |  | X |
| 2. Does the paper integrate gender theories in various parts? | X |  |
| 3. Does the paper develop gender concept and models? |  | X |
| 4. Does the paper interpret empirical findings from gender theories? |  | X |
| 5. Does the paper use gender theories in order to understand health problems? | X |  |
| 6. Does the paper use the health problem in order to illustrate that the theory is valid/ the implications of the theory? |  | X |
| 7. Do the authors use/ integrate gender theories in traditional gender blind theories? | X |  |
| 8. Do they use gender theory to criticise other feminist theories? | X |  |

| **22** | **Tolhurst et al 2012** |
| --- | --- |
| 1 | Post-modern and post-colonial feminism  Hegemonic masculinities  Gender mainstream  Intersectionality |
| 2 | “international health” |
| 3 | Neither a nor b. |

| Step 2 | Yes | No |
| --- | --- | --- |
| 1. Does the paper use gender theories in order to test hypothesis? |  | X |
| 2. Does the paper integrate gender theories in various parts? | X |  |
| 3. Does the paper develop gender concept and models? |  | X |
| 4. Does the paper interpret empirical findings from gender theories? |  | X |
| 5. Does the paper use gender theories in order to understand health problems? |  | X |
| 6. Does the paper use the health problem in order to illustrate that the theory is valid/ the implications of the theory? |  | X |
| 7. Do the authors use/ integrate gender theories in traditional gender blind theories? |  | X |
| 8. Do they use gender theory to criticise other feminist theories? | X |  |

| **23** | **Weber et al 2012** |
| --- | --- |
| 1 | Feminist intersectional framework  Response to CSDH, wants to ‘explore the role of power relations |
| 2 | Health well-being |
| 3 | 3a. Use intersectionality in order to analyse health  3b Use health in order to illustrate that the theory is valid. Conclude that the results “exemplify the ways in which social relations of power and control contribute to health inequities” (page 1833). |

| Step 2 | Yes | No |
| --- | --- | --- |
| 1. Does the paper use gender theories in order to test hypothesis? |  | X |
| 2. Does the paper integrate gender theories in various parts? | X |  |
| 3. Does the paper develop gender concept and models? |  | X |
| 4. Does the paper interpret empirical findings from gender theories? | X |  |
| 5. Does the paper use gender theories in order to understand health problems? | X |  |
| 6. Does the paper use the health problem in order to illustrate that the theory is valid/ the implications of the theory? | X |  |
| 7. Do the authors use/ integrate gender theories in traditional gender blind theories? |  | X |
| 8. Do they use gender theory to criticise other feminist theories? |  | X |

**Scandinavian journal of work and environmental health 2009**

| **24** | **Härenstam A 2009** |
| --- | --- |
| 1 | Gender order  Gendered contexts |
| 2 | “Health” |
| 3 | Uses health in order to illustrate that the theory and method are valid |

| Step 2 | Yes | No |
| --- | --- | --- |
| 1. Does the paper use gender theories in order to test hypothesis? |  | X |
| 2. Does the paper integrate gender theories in various parts? | X |  |
| 3. Does the paper develop gender concept and models? |  | X |
| 4. Does the paper interpret empirical findings from gender theories? |  | X |
| 5. Does the paper use gender theories in order to understand health problems? |  | X |
| 6. Does the paper use the health problem in order to illustrate that the theory is valid/ the implications of the theory? | X |  |
| 7. Do the authors use/ integrate gender theories in traditional gender blind theories? |  | X |
| 8. Do they use gender theory to criticise other feminist theories? |  | X |

INT REV PSYCHIATRY 2010

| **25** | **Andermann L 2010** | | |
| --- | --- | --- | --- |
| 1 | Social constructivism, universality of women | | |
| 2 | Mental health | | |
| 3 | 3a: uses gender theories in order to understand mental health | | |
|  | | | |
| Step 2 | | Yes | No |
| 1. Does the paper use gender theories in order to test hypothesis? | |  | X |
| 2. Does the paper integrate gender theories in various parts? | | X |  |
| 3. Does the paper develop gender concept and models? | |  | X |
| 4. Does the paper interpret empirical findings from gender theories? | |  | X |
| 5. Does the paper use gender theories in order to understand health problems? | | X |  |
| 6. Does the paper use the health problem in order to illustrate that the theory is valid/ the implications of the theory? | |  | X |
| 7. Do the authors use/ integrate gender theories in traditional gender blind theories? | |  | X |
| 8. Do they use gender theory to criticise other feminist theories? | |  | X |

ERGONOMICS

| **26** | **Ahlgren et al** | | |
| --- | --- | --- | --- |
| 1 | Multiple role theory, Role stress theory, gendered life circumstances | | |
| 2 | musculoskeletal disorders and emotional exhaustion | | |
| 3 | 3a Use gender concepts/theories in order to explain their findings | | |
|  | | | |
| Step 2 | | Yes | No |
| 1. Does the paper use gender theories in order to test hypothesis? | |  | X |
| 2. Does the paper integrate gender theories in various parts? | | X |  |
| 3. Does the paper develop gender concept and models? | |  | X |
| 4. Does the paper interpret empirical findings from gender theories? | | X |  |
| 5. Does the paper use gender theories in order to understand health problems? | | X |  |
| 6. Does the paper use the health problem in order to illustrate that the theory is valid/ the implications of the theory? | |  | X |
| 7. Do the authors use/ integrate gender theories in traditional gender blind theories? | |  | X |
| 8. Do they use gender theory to criticise other feminist theories? | |  | X |

SOC SCI MED 2012 SUICIDE

| **27** | **Canetto SS, Cleary A 2012** |
| --- | --- |
| 1 | Masculinities, gender as performative, critics a sex-difference framework and essentialism  Introduce gender theories which are used in the special issue. |
| 2 | Suicidal behaviour |
| 3 | Editorial, not relevant to code. |
|  | |

| **28** | **Scourfield J, Fincham B, Langer S, Shiner M. 2012** | | |
| --- | --- | --- | --- |
| 1 | Gendered identities and practices, masculinity crisis, objectivist rather than constructivist understanding | | |
| 2 | Suicide | | |
| 3 | Neither a, nor b | | |
|  |  | | |
| Step 2 | | Yes | No |
| 1. Does the paper use gender theories in order to test hypothesis? | |  | X |
| 2. Does the paper integrate gender theories in various parts? | | X |  |
| 3. Does the paper develop gender concept and models? | |  | X |
| 4. Does the paper interpret empirical findings from gender theories? | |  | X |
| 5. Does the paper use gender theories in order to understand health problems? | |  | X |
| 6. Does the paper use the health problem in order to illustrate that the theory is valid/ the implications of the theory? | |  | X |
| 7. Do the authors use/ integrate gender theories in traditional gender blind theories? | |  | X |
| 8. Do they use gender theory to criticise other feminist theories? | |  | X |

| **29** | **Adinkrah M 2012** | | |
| --- | --- | --- | --- |
| 1 | Masculinity, patriarchal structures, biological distinction between male and female human being, | | |
| 2 | Suicidal behaviour | | |
| 3 | Gender theories are used in order to understand the findings | | |
|  | | | |
| Step 2 | | Yes | No |
| 1. Does the paper use gender theories in order to test hypothesis? | |  | X |
| 2. Does the paper integrate gender theories in various parts? | | X |  |
| 3. Does the paper develop gender concept and models? | |  | X |
| 4. Does the paper interpret empirical findings from gender theories? | | X |  |
| 5. Does the paper use gender theories in order to understand health problems? | | X |  |
| 6. Does the paper use the health problem in order to illustrate that the theory is valid/ the implications of the theory? | |  | X |
| 7. Do the authors use/ integrate gender theories in traditional gender blind theories? | |  | X |
| 8. Do they use gender theory to criticise other feminist theories? | |  | X |

| **30** | **Mac An Ghaill M, Haywood C 2012.** | | |
| --- | --- | --- | --- |
| 1 | Differences within the group of men  Masculinities in violence | | |
| 2 | Suicide | | |
| 3 | The findings are interpreted/developed from gender theories and discussed in relation to health (suicide). But the findings do not include health. Thus, neither 3a nor 3b. | | |
|  |  | | |
| Step 2 | | Yes | No |
| 1. Does the paper use gender theories in order to test hypothesis? | |  | X |
| 2. Does the paper integrate gender theories in various parts? | | X |  |
| 3. Does the paper develop gender concept and models? | |  | X |
| 4. Does the paper interpret empirical findings from gender theories? | |  | X |
| 5. Does the paper use gender theories in order to understand health problems? | |  | X |
| 6. Does the paper use the health problem in order to illustrate that the theory is valid/ the implications of the theory? | |  | X |
| 7. Do the authors use/ integrate gender theories in traditional gender blind theories? | |  | X |
| 8. Do they use gender theory to criticise other feminist theories? | |  | X |

| **31** | **Cleary A 2012** | | |
| --- | --- | --- | --- |
| 1 | Constructions of masculinities, socialization of boys. Criticism against Durkheim, construction of men as one single group, of a gender difference approach, of Western dualism of body and mind | | |
| 2 | Suicide | | |
| 3 | 3a: Uses gender theories to understand the findings | | |
|  |  | | |
| Step 2 | | Yes | No |
| 1. Does the paper use gender theories in order to test hypothesis? | |  | X |
| 2. Does the paper integrate gender theories in various parts? | | X |  |
| 3. Does the paper develop gender concept and models? | |  | X |
| 4. Does the paper interpret empirical findings from gender theories? | |  | X |
| 5. Does the paper use gender theories in order to understand health problems? | | X |  |
| 6. Does the paper use the health problem in order to illustrate that the theory is valid/ the implications of the theory? | |  | X |
| 7. Do the authors use/ integrate gender theories in traditional gender blind theories? | |  | X |
| 8. Do they use gender theory to criticise other feminist theories? | | X |  |

| **32** | **Oliffe JL, Ogrodniczuk JS, Bottorff JL, Johnson JL, Hoyak K. 2012.** | | |
| --- | --- | --- | --- |
| 1 | Masculinity (identities, roles, norms, hegemonic), plurality of masc., agency within structure | | |
| 2 | Suicide | | |
| 3 | 3a. Gender theories were used in order to understand suicides | | |
|  | |  |  |
| Step 2 | | Yes | No |
| 1. Does the paper use gender theories in order to test hypothesis? | |  | X |
| 2. Does the paper integrate gender theories in various parts? | | X |  |
| 3. Does the paper develop gender concept and models? | |  | X |
| 4. Does the paper interpret empirical findings from gender theories? | | X |  |
| 5. Does the paper use gender theories in order to understand health problems? | | X |  |
| 6. Does the paper use the health problem in order to illustrate that the theory is valid/ the implications of the theory? | |  | X |
| 7. Do the authors use/ integrate gender theories in traditional gender blind theories? | |  | X |
| 8. Do they use gender theory to criticise other feminist theories? | |  | X |

| **33** | **Alston M 2012** | | |
| --- | --- | --- | --- |
| 1 | Gender relations, construction of masculinities, gendered life circumstances, hegemonic masculinity (male dominance), subordinated women, gender roles, | | |
| 2 | Suicide | | |
| 3 | 3a Understanding health among rural men requires gender theories. | | |
|  | | | |
| Step 2 | | Yes | No |
| 1. Does the paper use gender theories in order to test hypothesis? | |  | X |
| 2. Does the paper integrate gender theories in various parts? | | X |  |
| 3. Does the paper develop gender concept and models? | |  | X |
| 4. Does the paper interpret empirical findings from gender theories? | | X |  |
| 5. Does the paper use gender theories in order to understand health problems? | | X |  |
| 6. Does the paper use the health problem in order to illustrate that the theory is valid/ the implications of the theory? | |  | X |
| 7. Do the authors use/ integrate gender theories in traditional gender blind theories? | |  | X |
| 8. Do they use gender theory to criticise other feminist theories? | |  | X |
